# Differentiated Embedded Pilot Assisted Automatic Modulation Classification for OTFS System: A Multi-Domain Fusion Approach

**DOI:** 10.3390/s25144393

**Published:** 2025-07-14

**Authors:** Zhenkai Liu, Bibo Zhang, Hao Luo, Hao He

**Affiliations:** Ocean College, Jiangsu University of Science and Technology, Zhenjiang 212100, China; lzk04216@stu.just.edu.cn (Z.L.); luohao@just.edu.cn (H.L.); haohe@just.edu.cn (H.H.)

**Keywords:** automatic modulation classification, orthogonal time–frequency space, multi-domain fusion, embedded pilot

## Abstract

Orthogonal time–frequency space (OTFS) modulation has emerged as a promising technology to alleviate the effects of the Doppler shifts in high-mobility environments. As a prerequisite to demodulation and signal processing, automatic modulation classification (AMC) is essential for OTFS systems. However, a very limited number of works have focused on this issue. In this paper, we propose a novel AMC approach for OTFS systems. We build a dual-stream convolutional neural network (CNN) model to simultaneously capture multi-domain signal features, which substantially enhances recognition accuracy. Moreover, we propose a differentiated embedded pilot structure that incorporates information about distinct modulation schemes to further improve the separability of modulation types. The results of the extensive experiments carried out show that the proposed approach can achieve high classification accuracy even under low signal-to-noise ratio (SNR) conditions and outperform the state-of-the-art baselines.

## 1. Introduction

With the rapid development of wireless communication networks toward high-frequency bands and high-mobility scenarios, the bottlenecks of orthogonal frequency division multiplexing (OFDM) brought about by inter-carrier interference and Doppler spread have become increasingly prominent [1]. orthogonal time–frequency space (OTFS) modulation, proposed as a novel multi-carrier modulation scheme [2] and emerging as a key candidate technology for next-generation 6G mobile communication systems [3,4], can serve as a solution. By mapping information symbols to the delay-Doppler (DD) domain, OTFS systems effectively convert doubly dispersive channels into approximately quasi-static channels in the DD domain, thereby mitigating the time-selective fading caused by Doppler shifts in high-mobility environments [5]. This distinctive advantage leads OTFS to demonstrate remarkable potential in dynamic communication scenarios, including satellite communications (SATCOM) [6,7,8], unmanned aerial vehicle (UAV) communications [9,10], vehicle-to-everything (V2X) networks [11,12,13], etc.

Automatic modulation classification (AMC), a fundamental technology in wireless communications, aims to identify the modulation types of received signals without prior information, providing essential support for subsequent demodulation and signal processing [14]. Traditional AMC approaches primarily fall into two categories: likelihood-based AMC approaches [15,16] and feature-based AMC approaches [17,18]. While achieving theoretical optimality, maximum likelihood-based AMC can suffer from prohibitive computational complexity in model parameter estimation [19]. In contrast, feature-based AMC recognizes modulation schemes by leveraging characteristics such as high-order spectrum features, frequency spectrum signatures, and power spectrum attributes. In recent years, deep learning has achieved breakthroughs in different areas, e.g., image classification, speech recognition, etc. Driven by these developments, increasing research efforts have focused on integrating deep learning with modulation recognition [20]. Some works leverage convolutional neural networks to extract 2D signal features [21,22], some works design long short-term memory (LSTM)-based networks to capture the temporal features of signals [23,24], and a large portion of works build hybrid CNN–LSTM models that are simultaneously based on features from multiple dimensions [25,26,27,28]. There are other works that develop ResNet-based hybrid neural network models [29,30] and multi-stream/multi-scale fusion models [28,31].

Despite these existing AMC approaches, there are very few studies focusing on AMC for OTFS systems. Additionally, it is infeasible to directly apply the existing AMC approaches, which are primarily designed for OFDM systems, to OTFS systems and achieve satisfactory classification performance [32]. An AMC approach for OTFS systems is proposed in [33] by designing a hybrid CNN and LSTM network with a residual stack to process I/Q symbols. However, this method primarily leverages the time-domain features of the received signal and does not consider the intrinsic DD domain of OTFS signals.

In this paper, we investigate the AMC issue for OTFS systems by simultaneously taking into account features of the signals in both the time domain and delay-Doppler domain. To further boost the classification accuracy, we leverage embedded pilot patterns in the delay-Doppler plane to enhance the feature discriminability among modulation schemes. Embedded pilot structures have predominantly been adopted in channel estimation in OTFS systems [34,35]. However, to date, there are no works that harness it well for modulation recognition/classification.In this work, we develop and incorporate a differentiated pilot insertion scheme in AMC that leverages the inherent properties of OTFS pilot structures and introduces modulation-specific patterns to enhance modulation discriminability. In a nutshell, our contributions are summarized as follows:

- We propose a multi-domain fusion-based AMC approach for OTFS systems by designing a dual-stream CNN architecture that simultaneously incorporates the time-domain and DD-domain features of OTFS signals.
- We develop a differentiated embedded pilot insertion scheme which incorporates modulation-related pilot symbols in DD plane structure to enhance classification accuracy.
- We conduct extensive experiments, and the results demonstrate that the proposed approach can achieve high classification accuracy in high-mobility scenarios and low-signal-to-noise ratio (SNR) conditions and outperform the state-of-the-art approaches.

The remaining parts of this paper are organized as follows: Section 2 reviews the related literature and is followed by Section 3, which depicts the system model and describes the problem to be solved. In Section 4, the proposed method is elaborated on. Section 5 showcases and analyzes the numerical results. In Section 6, we conclude the paper with some final remarks.

## 2. Related Work

**Figure 1:**
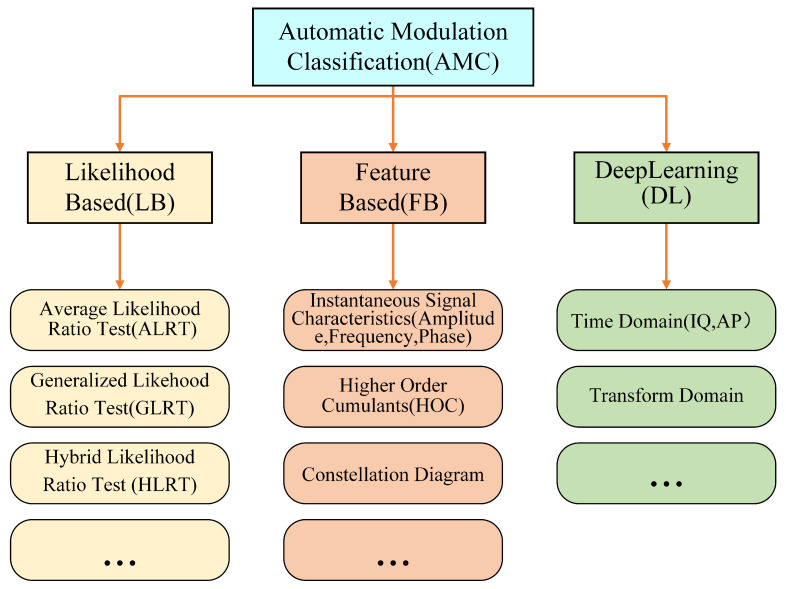
Automatic modulation classification methods.

Automatic modulation classification (AMC) plays a vital role in radio monitoring and spectrum management and has been widely applied in various communication systems, such as satellite communications and network communications. AMC was first proposed by researchers from Stanford University [36], and it aimed at overcoming the limitations of traditional manual identification methods and achieving high accuracy. Recent years have witnessed the development of different AMC methods, which can be generally categorized into three types: the likelihood-based method, the feature-based method, and the deep learning-based method (as shown in Figure 1).

**Likelihood-based method.** The modulation recognition method based on likelihood ratio testing (LRT) is carried out on top of composite hypothesis testing. First, the maximum likelihood function of the unknown signal and the optimal decision threshold are determined based on comprehensive analysis of signal characteristics. Then, the extracted statistics are compared with the thresholds to achieve modulation signal classification. The authors in [37] propose a modulation recognition method based on the Average Likelihood Ratio Test (ALRT), which shows potential in distinguishing between binary phase-shift keying (BPSK) and quadrature phase shift keying (QPSK) signals. In [38], a Generalized Likelihood Ratio Test (GLRT)-based AMC approach is proposed by combining power series with likelihood functions. It achieves comparable recognition performance to the average likelihood approach while reducing computational complexity and decreasing implementation difficulties. A Hybrid Likelihood Ratio Test (HLRT) classification approach, proposed in [39], both preserves the advantages of conventional algorithms and suppresses inter-symbol interference while maintaining reasonable computational complexity. Although likelihood-based methods can achieve optimal classification performance in the Bayesian sense, their practical application faces challenges due to the need to account for unknown parameters. As the number of unknown parameters increases, they become increasingly difficult to implement and computationally intensive.

**Feature based method.** This type of methods extracts key features from modulated signal samples and builds classifiers for modulation classification. As different types of signals are characterized by diverse features, adopting different types of features can significantly impact signal recognition accuracy. Typically, expertise-based features are categorized into time-domain features, cumulant-based features, spectral features, etc. To name a few, the authors in [40] extract and analyze nine distinct signal features, including amplitude, phase, and instantaneous frequency characteristics, and conduct modulation recognition based on these features. Ref. [41] utilizes fourth-order cumulants as signal features and employs a hierarchical model to progressively classify modulation types, including M-ary phase-shift keying (MPSK), M-ary quadrature amplitude modulation (MQAM), and M-ary pulse-amplitude modulation (MPAM). In [42], a hidden Markov model (HMM) approach is employed to analyze spectral correlation characteristics in the cyclic frequency domain. The feature-based methods can achieve relatively satisfactory recognition performance. However, they often require extensive preprocessing procedures and highly rely on feature extraction. When the received signal is contaminated by noise, the feature extraction process becomes less effective, leading to a degraded overall recognition performance.

**Deep learning-based method.** While it fundamentally belongs to the category of feature-based methods, we categorize it separately because of its prevalence in the existing work and significant methodological and conceptual differences from conventional feature-based methods.On one hand, the existing DL-based methods can be classified into time-domain waveform-based ones and transform-domain image-based ones, according to the types of data fed into the neural networks. On the other, they can be classified based on the different neural network architectures that they are built on. In particular, long short-term memory (LSTM) networks are employed to extract temporal features from input signals to improve classification accuracy [23,24]. Convolutional neural network (CNN)-based automatic modulation classification approaches are proposed in [21,22]. The authors in [25,27] propose hybrid CNN–LSTM models combining CNN’s spatial feature extraction and RNN’s temporal sequence processing capabilities for time-dependent signals. Ref. [26] adapts a convolutional long short-term deep neural network (CLDNN) to AMC issues, while [28] further proposes a multi-channel CLDNN (MCLDNN) approach. Leveraging the residual network (ResNet), a parallel architecture integrating residual network (ResNet) modules with gated recurrent units (GRUs) and cascaded LSTM networks with two-layer ResNets is developed in [29,30]. A three-stream deep learning framework is designed in [28], leveraging the independent/joint features of in-phase/quadrature (I/Q) components through multi-channel inputs to enhance spatiotemporal feature fusion. In [31], modulation classification is carried out by adopting a multi-channel input strategy and building a multi-scale neural network (MSNN) with multi-head self-attention and bidirectional GRUs.

The above existing works show good examples of AMC. However, the number of works focusing on AMC for OTFS systems is very limited. In particular, although existing deep learning-based works demonstrate the power of applying deep learning techniques in the field of AMC, determining how to leverage deep neural networks to enhance the accuracy of AMC for OTFS is still an urgent issue to tackle. In [33], an AMC approach for OTFS systems is proposed by designing a hybrid CNN and LSTM network with a residual stack to process I/Q symbols. However, it primarily relies on time-domain features of the received signal, while the intrinsic DD-domain features of OTFS signals remain unexploited. Moreover, although embedded-pilots are harnessed in channel estimation in OTFS systems [34,35], as far as we are aware, there is no AMC-related work incorporating them to boost the performance.In this paper, we propose an AMC approach for OTFS systems by building a dual-stream CNN-based framework to incorporate both time-domain and DD-domain information and by developing a differentiated embedded pilot insertion scheme. The experimental results demonstrate that the proposed approach can achieve high accuracy even under extreme conditions.

## 3. System Model

**Figure 2:**
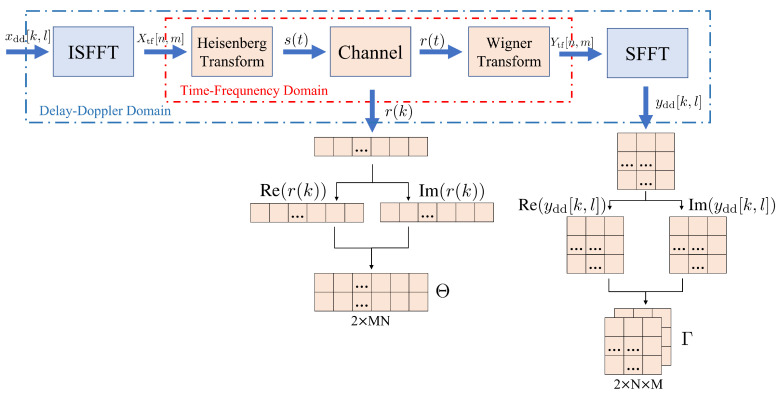
System model and acquisition of the time and delay-Doppler domain data.

We investigate the modulation recognition of the OTFS transmission system depicted in Figure 2. The information symbols $\left\{x_{\mathrm{dd}}\left[k,l\right],k=0,\ldots ,N-1,l=0,\ldots ,M-1\right\}$ are in a two-dimensional DD grid of size N×M, where *N* and *M* are the numbers of indices along the Doppler and delay axes, respectively. The symbol $x_{\mathrm{dd}}\left[k,l\right]$ is transformed to the TF-domain symbol $X_{\mathrm{tf}}\left[n,m\right]$, n=0,…,N−1, m=0,…,M−1 through the inverse symplectic finite Fourier transform (ISFFT) [43]:

(1) $$X_{\mathrm{tf}}\left[n,m\right]=\frac{1}{\sqrt{NM}}\sum_{k=0}^{N-1}\sum_{l=0}^{M-1}x_{\mathrm{dd}}\left[k,l\right]\;e^{j2\pi \left(\frac{nk}{N}-\frac{ml}{M}\right)}.$$

The signal is transformed from the TF domain to the time domain via the Heisenberg transform [34], and the transmitted time-domain signal is

(2) $$s\left(t\right)=\sum_{n=0}^{N-1}\sum_{m=0}^{M-1}X_{\mathrm{tf}}\left[n,m\right]\;g_{\mathrm{tx}}\left(t-nT\right)\;e^{j2\pi m\Delta f(t-nT)},$$

where Δf is the subcarrier spacing, *T* is the symbol duration, and $g_{\mathrm{tx}}\left(t\right)$ denotes the transmit pulse shaping filter.

The signal transmitted through the channel is affected by the channel impulse response h(τ,ν) in the DD domain:

(3) $$h\left(\tau ,\nu \right)=\sum_{i=1}^{P}h_{i}\;\delta \left(\tau -\tau_{i}\right)\;\delta \left(\nu -\nu_{i}\right),$$

where *P* is the number of propagation paths, and $h_{i}$ is the gain of the *i*-th path. $\tau_{i}$ and $\nu_{i}$ respectively denote the delay and Doppler shift of the *i*-th path,

(4) $$\tau_{i}=\frac{l_{i}}{M\Delta f},\;\nu_{i}=\frac{k_{i}}{NT},$$

where $l_{i}$ is the delay index, and $k_{i}$ is the Doppler index.

Then, the received signal r(t) is

(5) $$r\left(t\right)=\;\int \int \;h\left(\tau ,\nu \right)s\left(t-\tau \right)e^{j2\pi \nu (t-\tau )}\;d\tau d\nu +\omega \left(t\right),$$

where ω(t) represents the additive white Gaussian noise.

The received symbols in the TF domain $Y_{\mathrm{tf}}\left[n,m\right]$ can be obtained via Wigner Transform:

(6) $$Y_{\mathrm{tf}}\left[n,m\right]=\int r\left(t\right)\;g_{\mathrm{rx}}^{*}\left(t-nT\right)\;e^{-j2\pi m\Delta f(t-nT)}\;dt.$$

Here, $g_{\mathrm{rx}}\left(t\right)$ denotes the pulse shaping filter at the receiver.

Finally, applying the symplectic finite Fourier transform (SFFT) to the samples yields the symbols in the DD domain [43]:

(7) $$y_{\mathrm{dd}}\left[k,l\right]=\frac{1}{\sqrt{NM}}\sum_{n=0}^{N-1}\sum_{m=0}^{M-1}Y_{\mathrm{tf}}\left[n,m\right]e^{-j2\pi \left(\frac{nk}{N}-\frac{ml}{M}\right)}.$$

In this paper, we propose an automatic modulation classification framework for OTFS systems as described above, which involves a differentiated embedded pilot insertion based DD plane structure, dataset construction scheme, and a dual-stream CNN architecture-based multi-domain fusion approach.

## 4. Proposed Method

In this section, we first develop a differentiated embedded pilot insertion scheme, and on top of that, propose a multi-domain fusion-based automatic modulation classification approach for OTFS systems by combining DD-domain and time-domain signals through a dual-stream CNN-based architecture.

### 4.1. Differentiated Embedded Pilot Insertion Scheme

**Figure 3:**
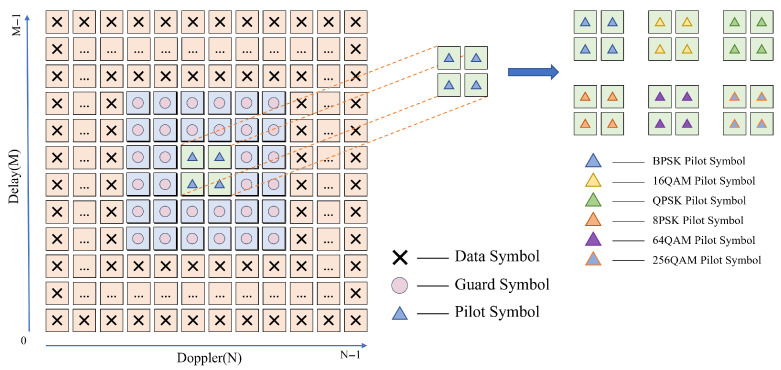
Differentiated embedded pilot insertion scheme.

We propose a differentiated embedded pilot insertion scheme that incorporates in the DD plane distinct pilot symbols related to modulation, which are surrounded by zero-padded guard bands, as illustrated in Figure 3. While preserving the inherent advantage of no interference between data and pilot signals as in existing pilot insertion schemes [34], it can further facilitate the neural network model designed hereafter to extract modulation-related features from the received signals.

Specifically, we employ differentiated pilot values for different modulation schemes. We define the set of modulation schemes as M={BPSK,QPSK,8PSK,16QAM,64QAM,256QAM} and the corresponding pilot set as $P={\left\{P_{M_{i}}\right\}}_{M_{i}\in M}$, where $P_{M_{i}}$ is the unique pilot symbol associated with modulation $M_{i}\in M$. The specific values for P depend on the channel types (e.g., PDCCH, PDSCH, PUCCH, PUSCH) and the utility of the reference signals (e.g., CSI-RS, SRS), and can be set according to rules indicated in Release 18 of the 3GPP NR specification [44].

In the embedded pilot design, the pilot symbols (i.e., P∈P) are placed at specific positions in the DD domain grid, and the guard symbols (i.e., 0) are configured around the pilots to isolate them from data symbols (i.e., *D*) [35]. This configuration can be expressed as

(8) $$x_{\mathrm{dd}}\left[k,l\right]=\left\{\begin{array}{cc} P, & \mathrm{if}\;\left(k,l\right)\in F_{p},\\ 0, & \mathrm{if}\;\left(k,l\right)\in G_{p},\\ D, & \mathrm{otherwise}, \end{array}\right.$$

where $F_{p}$ denotes the set of pilot positions in the DD grid, while $G_{p}$ represents the guard symbol region surrounding the pilot, and its size is constrained by the maximum delay and maximum Doppler shift.

The DD-domain structure based on the above differentiated embedded pilots can capture characteristics of different modulation schemes and provide additional information on modulation, thus allowing signals to be well-distinguished from each other. Therefore, the classification accuracy at the receiver can further be improved.

### 4.2. Dataset Design

In the OTFS system considered, the symbol is transformed from the DD domain to the time domain via ISFFT and Heisenberg transform. After the wireless channel transmission, the received signal is transformed into the DD domain via the Wigner transform and SFFT. We design a data acquisition scheme to extract two distinct data flows in the time and DD domains from the above process and employ them as the input to the neural network, as shown in Figure 2. We consider *M* subcarriers in the delay dimension and *N* symbols in the Doppler dimension.

We construct the time-domain data vector Θ with a dimension of 2×MN, using the in-phase (real) component and quadrature (imaginary) component of r[l] (l=0,…,MN−1), which is a discrete sampled version of the received time-domain signal r(t):

(9) Θ=[Re(r[l])]l∈[0,MN−1]Im(r[l])]l∈[0,MN−1]∈R2×MN.

To generate data for the DD-domain stream, we split the DD-domain symbol grid $y_{\mathrm{dd}}$ into an in-phase (real) component and a quadrature (imaginary) component with the dimensions of N×M and construct a DD-domain input data matrix Γ with a dimension of 2×N×M:

(10) Γ=[Re(ydd[k,l])]k∈[0,N−1],l∈[0,M−1]Im(ydd[k,l])]k∈[0,N−1],l∈[0,M−1]∈R2×N×M.

### 4.3. Dual-Stream Architecture for Multi-Domain Fusion

**Figure 4:**
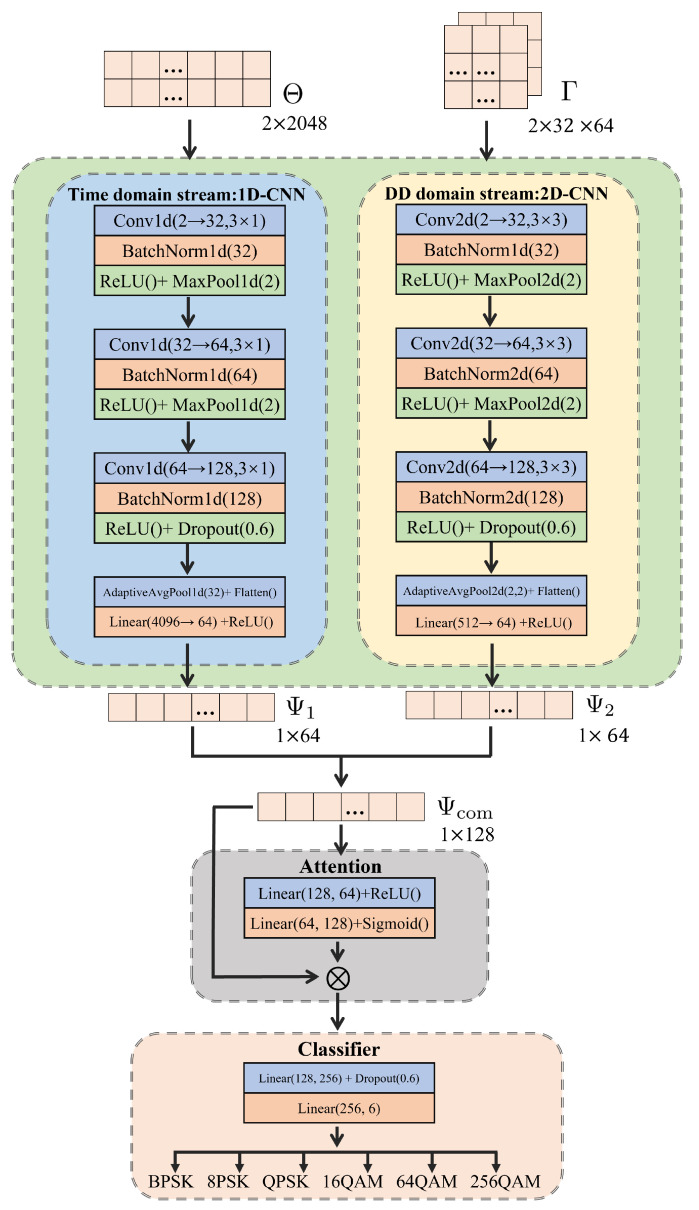
Dual-stream neural network architecture for multi-domain fusion.

We propose a multi-domain fusion approach for modulation classification by developing a dual-stream CNN-based architecture, which is characterized by the 1D-CNN branch and 2D-CNN branch for processing time-domain data and DD-domain data, as illustrated in Figure 4 (Note that the specific valuesblue, e.g., dropout rate of 0.6, adopted in the neural network architecture are based on numerical tests and comparisons carried out in the experiments. Please refer to Section 5 for more details). (empiracally considering N=32, M=64).

The 1D-CNN branch processes time-domain data (Θ) with an input shape of (2, 2048). The first block employs 32 kernels of 3×1 convolutions, followed by batch normalization and ReLU activation, and then performs 2× downsampling (2048→1024). The second block contains 64 convolutional kernels with the same processing flow (1024→512) thereafter. The third block uses 128 convolutional kernels and incorporates a dropout of rate 0.6. Finally, after transforming features to the dimensions of 32 via adaptive average pooling and passing through a fully connected layer, the branch outputs a 64-dimensional feature (i.e., $\Psi_{1}$).

The 2D-CNN branch processes delay-Doppler-domain data (Γ) with an input shape of (2, 32, 64). The first block uses 32 kernels of 3×3 convolutions, followed by batch normalization, ReLU activation, and pooling. The second block and third block respectively employ 64 and 128 convolutional kernels. Finally, the data passes through a 2×2 adaptive average pooling layer and a fully connected layer, and results in an output with a dimension of 64 (i.e., $\Psi_{2}$).

The features from 1D-CNN and 2D-CNN branches are combined, resulting in a 128-dimensional $\Psi_{\mathrm{com}}$, which is then fed into an attention module. This module generates adaptive weights that can be applied to $\Psi_{\mathrm{com}}$ to emphasize the most significant aspects in features. The final classifier comprises two fully connected layers, with a dropout rate of 0.6 applied between them to prevent overfitting.

### 4.4. Computational Complexity Analysis

The computational complexity of the proposed model is analyzed by examining neural network components. We define *B* as the batch size, $C_{in1}$ and $C_{in2}$ as the numbers of input channels for 1D and 2D branches, *L* the sequence length in the 1D branch, H×W as the spatial dimensions in the 2D branch, $K_{1},\;K_{2},\;K_{3}$ the sizes of kernels, $C_{out1},\;C_{out2},\;C_{out3}$ as the number of output channels of different stages, *N* as the number of output classification categories, $S_{1}$ and $S_{2}$ as the sizes after 1D-CNN and 2D-CNN feature extraction, $D_{att}$ as the attention dimension, and $D_{fc1}$ as the first fully connected layer dimension. Then, the complexity of the 1D-CNN branch, which processes time-domain signals with input shape $\left(B,C_{in1},L\right)$, is $O_{1D}\approx O\left(B\cdot L\cdot C_{in1}\cdot C_{out1}\cdot K_{1}\right)$, while the complexity of the 2D-CNN, which processes delay-Doppler images with input shape $\left(B,C_{in2},H,W\right)$, is $O_{2D}\approx O\left(B\cdot H\cdot W\cdot C_{in2}\cdot C_{out1}\cdot K_{1}^{2}\right)$. The complexity of the attention mechanism is $O_{Attention}\approx O\left(B\cdot C_{out3}\cdot D_{att}\right)$, and the complexity of the classifier module is $O_{Classifier}\approx O\left(B\cdot C_{out3}\cdot D_{fc1}\right)$. Therefore, the total complexity is $O_{model}=O_{1D}+O_{2D}+O_{Attention}+O_{Classifier}$, which can be approximately represented as $O_{model}\approx O\left(B\cdot L\cdot C_{in1}\cdot C_{out1}\cdot K_{1}\right)+O\left(B\cdot H\cdot W\cdot C_{in2}\cdot C_{out1}\cdot K_{1}^{2}\right)$, considering L≫max(H,W) in typical implementations.

## 5. Numerical Results

In this section, we evaluate the proposed differentiated embedded pilot and multi-domain fusion-based OTFS automatic modulation classification approach from diverse aspects. Extensive experiments are carried out to investigate the modulation classification accuracy w.r.t. different SNRs, the effects of pilot symbols, different NN architectures for time-domain and DD-domain data streams, and maximum Doppler shifts. Moreover, the proposed approach is compared with state-of-the-art baselines.

### 5.1. Experimental Settings and Performance Metric

The dataset consists of signals modulated by BPSK, QPSK, 8PSK, 16QAM, 64QAM, and 256QAM, with the SNRs ranging within {−5, 0, 5, 10, 15, 20, 25} dB. For each case in the combination set of such modulation schemes and SNR conditions, 2000 samples are generated; therefore, the complete dataset comprises 84,000 samples in total. To ensure effective model training and evaluation, a dataset is constructed with training, validation, and test subsets with the proportion of 7:2:1, respectively. The training phase spans 80 epochs with a batch size of 400 samples per iteration, optimized by the Adam algorithm with a learning rate of 0.0035. More experiment settings are listed in Table 1.

The experiments are conducted on a device with a single NVIDIA RTX 4090 GPU (24 GB), an Intel Xeon Gold 6430 CPU (16 cores), and 128 GB RAM. With the specified configurations, model training takes an average of approximately 7.2 min, with each epoch spanning an average of 5.4 s. With a trained model, it takes 0.054 ms for modulation classification of a signal. According to the above time costs, the proposed model can be implemented in real-time wireless operations. First, once trained, the model can be utilized for an extended period and does not need to be retrained unless the network undergoes significant changes. Second, 0.054 ms is an acceptable operation duration compared to the intervals (e.g., typically 0.1∼10 ms in different cases) between two modulation classifications in real wireless systems.

In the experiments, without loss of generality, the pilot values within the pilot matrix are kept identical for simplification. The values adopted for different modulation types are listed in Table 2.

The performance of modulation classification is evaluated based on the accuracy, precision, recall, and F1-score metrics. Let the entire sample set be divided into four subsets based on the true labels and predicted outcomes, i.e., the sets of true positives (TP), false positives (FP), true negatives (TN), and false negatives (FN); then, the four metrics are computed as follows:

The accuracy quantifies the proportion of correctly classified samples in the entire dataset and is defined by

(11) $$\mathrm{Accuracy}=\frac{\mathrm{TP}+\mathrm{TN}}{\mathrm{TP}+\mathrm{TN}+\mathrm{FP}+\mathrm{FN}}.$$

The precision measures the proportion of correctly predicted positive instances among all the predicted positive instances:

(12) $$\mathrm{Precision}=\frac{\mathrm{TP}}{\mathrm{TP}+\mathrm{FP}}.$$

The recall measures the proportion of correctly predicted positive instances among all the actual positive instances:

(13) $$\mathrm{Recall}=\frac{\mathrm{TP}}{\mathrm{TP}+\mathrm{FN}}.$$

The F1-score is the harmonic mean of precision and recall, providing a balanced evaluation metric:

(14) $$F1=2\times \frac{\mathrm{Precision}\times \mathrm{Recall}}{\mathrm{Precision}+\mathrm{Recall}}.$$

### 5.2. Performance Analysis

**Figure 5:**
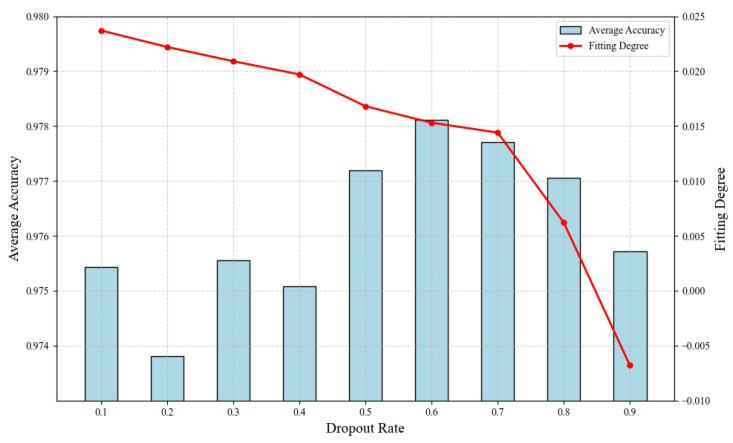
Accuracy and fitting degree across different dropout rates.

First of all, we evaluate the impact of the dropout rate on the model training in Figure 5. We measure fitting degree as the average difference between training set accuracy and validation set accuracy over the batches from convergence until the end of training. As can be seen, the model with the dropout rate of 0.6 reaches the best trade-off between classification accuracy and regularization effectiveness; it achieves highest average accuracy while effectively preventing overfitting and thus guaranteeing generalization. Therefore, a dropout rate of 0.6 is employed in the neural network model design.

**Figure 6:**
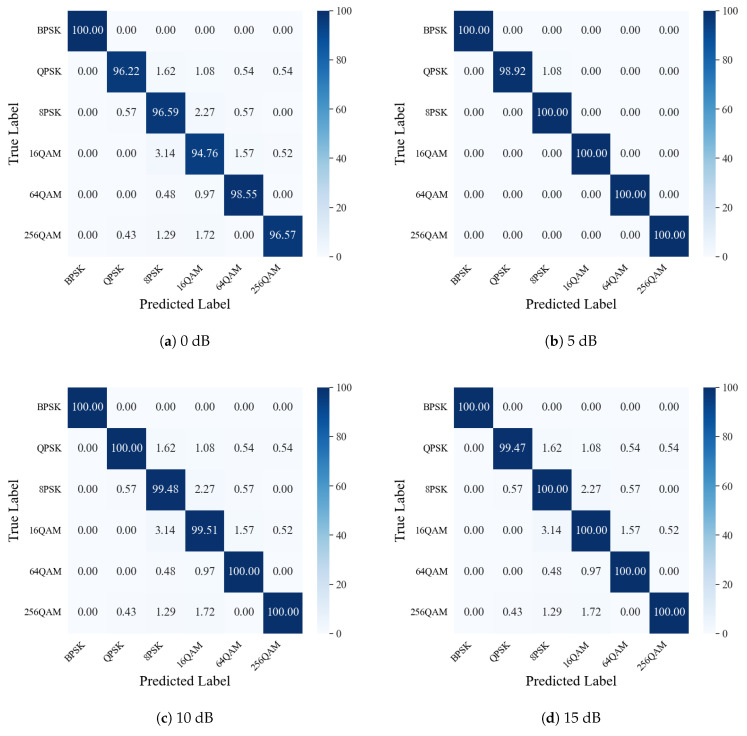
Confusion matrix of modulation classification results.

Figure 6 shows the confusion matrices of modulation classification at different SNRs ranging from 0 dB to 15 dB. As can be seen, the classification accuracy is significantly improved as the SNR is raised from 0 dB to 15 dB. Moreover, in Figure 6a,b, it is obvious that even under low SNR conditions (i.e., 0 dB and 5 dB), the confusion matrices exhibit apparent diagonal patterns, with 100% classification accuracy for some modulation schemes. Figure 6c,d further demonstrate that our approach shows excellent classification performance at high SNRs.

**Figure 7:**
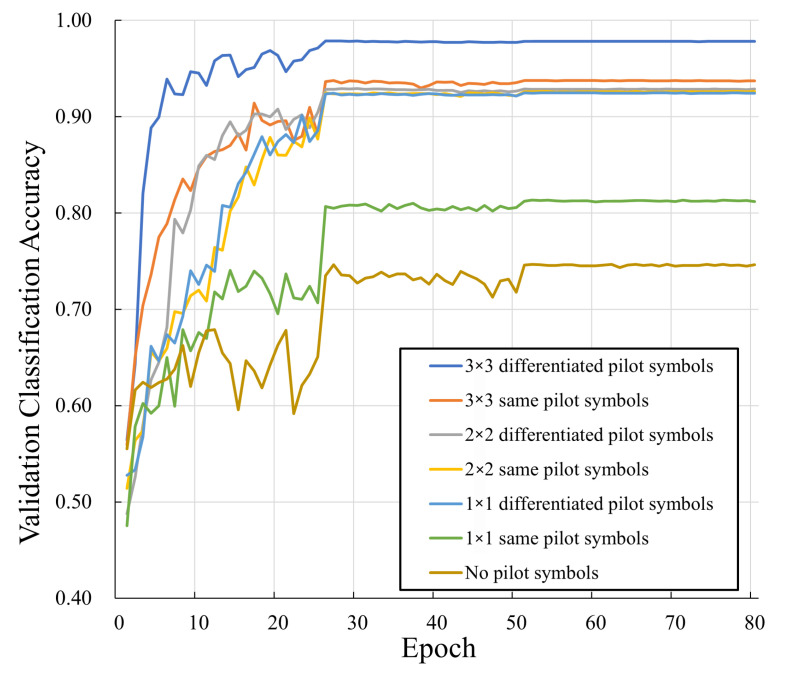
Comparison in terms of different pilot structures.

In Table 3 and Figure 7, we investigate the effect of the proposed differentiated embedded pilot insertion scheme on the neural network training and modulation classification performance. Compared with data structures using identical pilot symbols, the ones embedded with differentiated pilot values show higher classification accuracy thanks to the feature differences introduced among modulation schemes. Moreover, the number of embedded pilots also has an impact on the classification accuracy.

**Figure 8:**
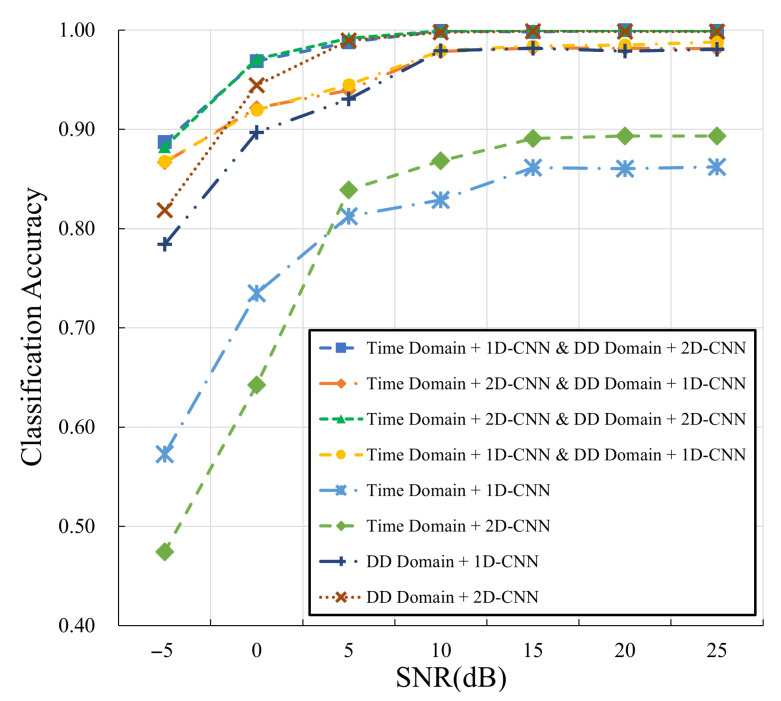
Comparison in terms of different NN architectures.

Furthermore, we conduct experiments for multi-domain and single-domain approaches whose results are shown in Figure 8 and Table 4. As can be seen, the DD domain NN branch, especially the one with the 2D-CNN, substantially contributes to a high modulation classification accuracy. Based on that, the incorporation of the time-domain NN branch can significantly boost the performance as the SNRs vary within [−5, 5] dB. This accounts for why the proposed combination of DD domain and time domain can achieve high classification accuracy under varying SNR conditions, including even −5 and 0 dB. Note that the structure (i.e., 1D-CNN/2D-CNN) of the time domain NN branch has limited effects on the performance; therefore, it is sufficient for the time-domain NN branch to adopt 1D-CNN for computational efficiency.

**Figure 9:**
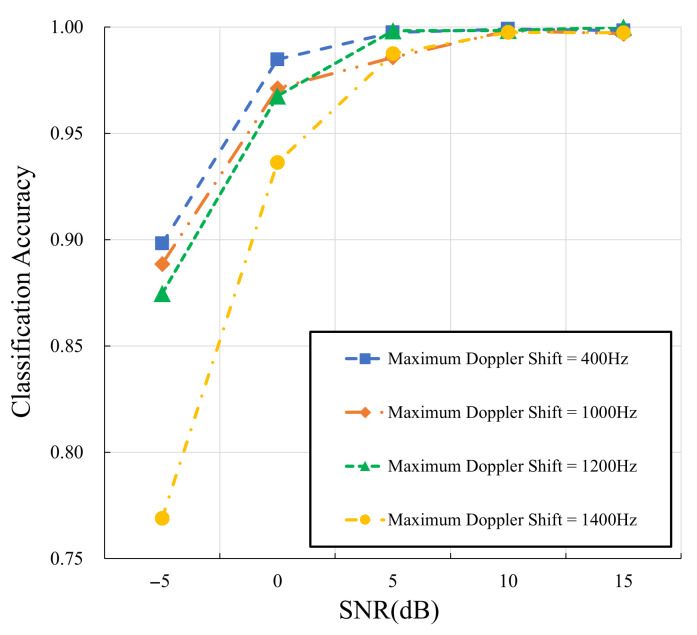
The performance of the proposed approach w.r.t. different maximum Doppler shifts.

The impact of maximum Doppler shifts on the classification performance of the proposed approach is investigated in Figure 9 and Table 5. The experiments are conducted with a carrier frequency of 5 GHz and maximum Doppler shifts of 400 Hz, 1000 Hz, 1200 Hz, and 1400 Hz, which correspond to the moving speeds of 86 km/h, 216 km/h, 260 km/h, and 302 km/h, covering common speed ranges of transportation. Therefore, the robustness of the proposed approach under various mobility conditions is comprehensively evaluated. Table 5 presents the classification accuracy, precision, recall, and F1-score over SNRs ranging from −5 dB to 25 dB under four different maximum Doppler shift conditions. As we can see, a high classification accuracy (approaching 75%) can be achieved even in a highly dynamic scenario (characterized by a maximum Doppler shift of 1400 Hz) with a low SNR (i.e., −5 dB), demonstrating the robustness of the proposed approach in extreme conditions.

**Figure 10:**
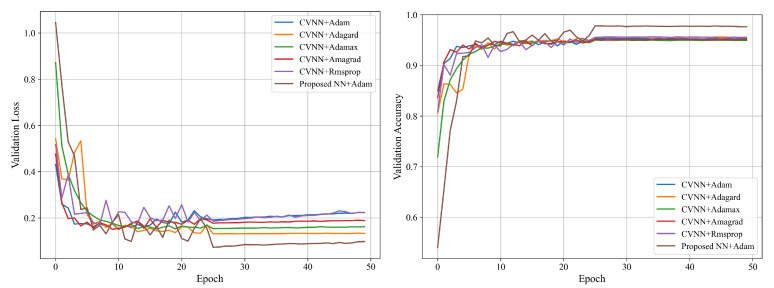
Comparison between CVNN models with different optimizers and the proposed approach.

As the received signal is inherently in the complex domain, in Figure 10, we compare the proposed approach, i.e., the proposed neural network (NN) architecture with Adam optimizer, with the complex-valued neural network (CVNN) model with different optimizers, including Adam, Adagrad, Adamax, Amagrad, and RMSprop, in terms of validation loss and accuracy. Apparently, when applied to CVNN, the Adagrad optimizer achieves lower validation loss and a faster convergence rate, while the Adam optimizer exhibits smoother curves with greater stability. Last but not least, the proposed approach can achieve the lowest loss and highest accuracy.

**Figure 11:**
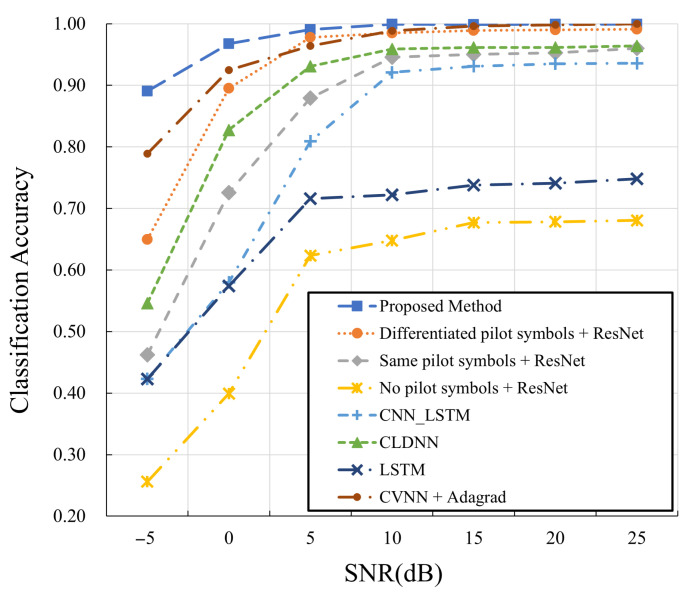
Comparison with state-of-the-art approaches.

In Figure 11, the proposed approach is compared with different state-of-the-art baseline approaches. ResNet [14], originally designed for automatic modulation classification in OFDM systems, is adapted to OTFS systems and evaluated upon datasets with differentiated pilots, identical pilots, and no pilots. The baselines CLDNN [26], LSTM [24], CNN_LSTM [33], and CVNN with Adagrad optimizer [45] adopt differentiated pilot structures. The results for ResNet [14] further demonstrate the advantage of the proposed differentiated pilots. Compared to all the state-of-the-art baselines, the proposed approach, characterized by the dual-stream network architecture, shows superior classification performance (at least 90% accuracy) under both low- and high-SNR conditions. Table 6 records average metrics including accuracy, precision, recall, and F1-score over all the SNR values. The near-identical values of the metrics for the proposed approach indicate that the generated AMC model prevents biases among classification tasks well.

## 6. Conclusions

In this paper, we have proposed a novel multi-domain fusion-based automatic modulation classification approach for OTFS systems. We have designed a new dual-stream CNN-based architecture that exploits both the time-domain and DD-domain signal features. Moreover, we have introduced a differentiated embedded pilot structure in the frame design, which incorporates the modulation-related symbol to further improve discriminability among different modulation schemes. Experimental results have demonstrated that the proposed approach can outperform the state-of-the-art baselines and achieve an average classification accuracy of 97.8% across a wide SNR range from −5 dB to 25 dB in high-mobility environments.

## Figures and Tables

**Table 1:** Simulation parameters.

Parameter	Value
Delay-Doppler grid size	N = 32, M = 64
Pilot symbol dimensions	3 × 3
Guard interval lengths	2
Sampling rate (kHz)	100
Maximum Doppler shift (Hz)	1000
Carrier frequency (GHz)	5
Channel model	Extended vehicular A model (EVA)
Modes of modulation	BPSK, QPSK, 8PSK, 16QAM, 64QAM, 256QAM

**Table 2:** Pilot signal configurations.

Modulation Type	Pilot Type	Value
BPSK	Real number	2
QPSK	Complex number	1+j
8PSK	Phase rotation	$e^{j\pi /4}$
16QAM	Complex number	1.5+1.5j
64QAM	Complex number	2+2j
256QAM	Real number	2.5

**Table 3:** Average classification accuracy under different pilot symbol configurations.

Pilot Symbol Configuration	Average Classification Acc. (%)
3 × 3 differentiated pilot symbols	97.8
3 × 3 same pilot symbols	93.7
2 × 2 differentiated pilot symbols	92.8
2 × 2 same pilot symbols	92.6
1 × 1 differentiated pilot symbols	92.4
1 × 1 same pilot symbols	81.2
No pilot symbols	74.6

**Table 4:** Average classification accuracies of hybrid 1D/2D CNN models for time and DD domains.

Parameter	Average Classification Acc. (%)	Parameter	Average Classification Acc. (%)
Time Domain + 1D-CNN & DD Domain + 2D-CNN	97.8	Time Domain + 1D-CNN	79.0
Time Domain + 2D-CNN & DD Domain + 1D-CNN	95.0	Time Domain + 2D-CNN	78.6
Time Domain + 2D-CNN & DD Domain + 2D-CNN	97.7	DD Domain + 1D-CNN	93.3
Time Domain + 1D-CNN & DD Domain + 1D-CNN	95.3	DD Domain + 2D-CNN	96.4

**Table 5:** Average classification performance under different maximum Doppler shifts.

Maximum Doppler Shift (Speed)	Accuracy (%)	Precision (%)	Recall (%)	F1-Score (%)
400 Hz (86 km/h)	98.26	98.27	98.26	98.27
1000 Hz (216 km/h)	97.81	97.81	97.81	97.81
1200 Hz (260 km/h)	97.71	97.77	97.73	97.75
1400 Hz (302 km/h)	95.41	95.43	95.40	95.40

**Table 6:** Classification Performance Metrics of Different Approaches.

Model	Accuracy (%)	Precision (%)	Recall (%)	F1-Score (%)
Proposed Method	97.81	97.81	97.81	97.81
Differentiated pilot symbols + ResNet [14]	92.50	92.57	92.52	92.54
Same pilot symbols + ResNet [14]	83.87	83.90	83.89	83.88
no pilot symbols + ResNet [14]	56.45	60.81	56.14	56.97
CNN_LSTM [33]	79.09	79.06	79.14	79.09
CLDNN [26]	87.87	88.84	87.87	87.81
LSTM [24]	66.60	69.30	59.50	64.00
CVNN + Adagrad [45]	95.62	95.64	95.63	95.63

## Data Availability

Dataset available on request from the authors.
